# Effect of Landscape Structure on Species Diversity

**DOI:** 10.1371/journal.pone.0066495

**Published:** 2013-06-19

**Authors:** Paulo R. A. Campos, Alexandre Rosas, Viviane M. de Oliveira, Marcelo A. F. Gomes

**Affiliations:** 1 Departamento de Física, Universidade Federal de Pernambuco, Recife, Pernambuco, Brazil; 2 Departamento de Física, Universidade Federal da Paraíba, João Pessoa, Paraíba, Brazil; 3 Departamento de Física, Universidade Federal Rural de Pernambuco, Recife, Pernambuco, Brazil; Universitat Pompeu Fabra, Spain

## Abstract

The effects of habitat fragmentation and their implications for biodiversity is a central issue in conservation biology which still lacks an overall comprehension. There is not yet a clear consensus on how to quantify fragmentation even though it is quite common to couple the effects of habitat loss with habitat fragmentation on biodiversity. Here we address the spatial patterns of species distribution in fragmented landscapes, assuming a neutral community model. To build up the fragmented landscapes, we employ the fractional Brownian motion approach, which in turn permits us to tune the amount of habitat loss and degree of clumping of the landscape independently. The coupling between the neutral community model, here simulated by means of the coalescent method, and fractal neutral landscape models enables us to address how the species–area relationship changes as the spatial patterns of a landscape is varied. The species–area relationship is one of the most fundamental laws in ecology, considered as a central tool in conservation biology, and is used to predict species loss following habitat disturbances. Our simulation results indicate that the level of clumping has a major role in shaping the species–area relationship. For instance, more compact landscapes are more sensitive to the effects of habitat loss and speciation rate. Besides, the level of clumping determines the existence and extension of the power-law regime which is expected to hold at intermediate scales. The distributions of species abundance are strongly influenced by the degree of fragmentation. We also show that the first and second commonest species have approximately self-similar spatial distributions across scales, with the fractal dimensions of the support of the first and second commonest species being very robust to changes in the spatial patterns of the landscape.

## Introduction

There is an overwhelming belief that habitat loss and fragmentation are the major forces contributing to the decline of biological diversity, which is supported by both theoretical and empirical studies [1]–[4]. On the other hand, there is also controversial evidence, based on long-term empirical surveys that provide instances of a positive correlation between species richness and habitat loss and fragmentation [5]–[7]. In this context, there is an extremely fruitful debate about the relative significance of habitat loss and changes in spatial configuration on the outcome of the spatial patterns of species distribution in fragmented landscapes [8].

One important tool to address this question is the species–area relationship (SAR), which has reached the status of an ecological law [9]. The species–area relationship describes the increase in the number of species along a gradient of ecosystems of increasing size. It has become a nearly ubiquitous pattern of biodiversity. The SAR has a major role in conservation biology, since it can be used as an indirect method to predict extinction rates from habitat loss [10], [11]. To study how habitat loss and fragmentation influence the scenarios displayed by the species–area relationships we make use of methods of modelling neutral landscapes, which can capture the essence of the spatial patterns of real landscapes [12]. In natural landscapes, habitats tend to be clumped or spatially correlated, and therefore the percolation maps [12], [13], whereby completely random landscape strucutures are produced, do not provide a satisfactory means for assessing the ecological consequences of a landscape structure because it is not possible to disentangle the effects of amount of habitat from those of its fragmentation [12], [14]. To circumvent this limitation, here the landscape is modelled through the use of an important class of neutral landscape models, known as fractal landscapes [15], [16].

In this paper the diversity of species is investigated in a spatially explicit neutral community using a model similar to the voter model of Durrett and Levin [17], [18]. The voter model with mutation, as defined by Durrett and Levin [18], is one of the first and simplest spatially explicit models for studying the observed patterns of the species–area relationships. The model is represented on a two-dimensional lattice, where every site is occupied by a single individual. The dynamics of the model accounts for the processes of birth, local dispersal and speciation, the latter resulting from occurrences of point mutation. One important feature of the model is the functional equivalence between different species, which is one of the basic assumption of Hubbell’s neutral theory [19]. This means that the rates of death, birth, dispersal and speciation are the same regardless of the individual’s identity. Unlike the original proposal here we consider fragmented landscapes, where some sites of the lattice can be unsuitable for occupancy. In addition, the configuration and distribution of suitable/unsuitable habitats are controlled by a fractional Brownian motion, which produces spatially correlated landscapes. In this generalized model, we can easily tune the level of spatial autocorrelation and so produce very distinct landscape structures.

This paper also presents some simulation results for species abundance distributions and the fractal dimension of the support for where the first and second commonest species reside. In what follows, we describe our modelling in the section Material and Methods, and then we discuss the results. We present our conclusions in the last section.

## Materials and Methods

Our study is based on extensive computer simulations. Because we are only interested in the spatial patterns of species distribution in an equilibrium state, a powerful coalescence method which employs ideas from coalescence theory [20], [21] fulfills the requirements of computer efficiency and more importantly the attainment of an equilibrium regime. A brief description of this method follows.

### The Model

The model assumes a finite population of size 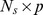 arranged on a two-dimensional square lattice with sides of length 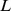, so that 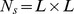. The proportion of suitable habitats 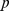 is an input parameter of the model. In this way, each cell (habitat) of the lattice can be occupied by at most a single individual. Unsuitable habitats are considered to be uninhabitable and remain unoccupied indefinitely. Of course, this is an oversimplication because habitats that are hostile to one set of species can provide a beneficial environment to another set of species. Multi-habitat landscape models can be used in a more generalized context since they take into account the affinities of each species to the different types of habitat [22], [23]. Nevertheless, our framework assumes a complete equivalence between the species, which is a premise of the neutral theory [19].

The distribution of suitable/unsuitable cells is not completely random but has a controlled level of aggregation, a desirable feature of more realistic spatial patterns for habitat distribution [24]. With this aim, we use fractal landscapes, a long standing and useful tool in realistic rendering and modelling of geographical reliefs and frontiers, among other natural surfaces and interfaces [15]. Once the landscape is established, the next step concerns the dynamics of the neutral community model.

### Fractal Landscapes

Fractal landscapes are constructed through the use of fractional Brownian motion [25]. A fractional Brownian motion (fBm) is a generalization of a random process 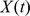 with Gaussian increments so that
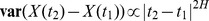
(1)where 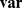 denotes the variance, and 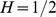 characterizes the ordinary Brownian motion. In fractional Brownian motion the scaling behaviour of the different traces is determined by the Hurst exponent, 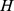, taken in the range 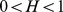. When 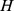 is around zero, the fBm produces very rough structures, while at the other extreme 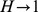, smooth traces are generated. Thus, by varying the parameter 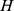 starting from zero, one produces a fragmentation gradient, and the landscape structure changes gradually from highly fragmented to highly clumped. The fBm conceives statistically self-affine structures with the symmetry property 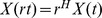 for any real 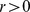 [15]. To generate an fBm we employ the spectral synthesis method, which is established on the spectral representation of the samples of the process 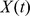. The spectral density of a fractional Brownian motion depends on the frequency 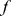 according to
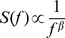
(2)where 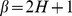. In order to convert the resulting structure into two-dimensional spatial patterns of ones and zeros corresponding to suitable and unsuitable habitats, the fBm is segmented [16]. The points which are above (below) a given critical elevation 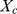 are ascribed the value one (zero). The critical elevation 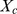 is settled by the fraction of suitable habitats 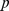. Thus, each realization of the landscape construction corresponds to a new value of 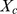.

### Neutral Dynamics

The species–area relationship (SAR) is a central concept in ecology and has acquired the status of an ecological law [9], [26]. It has become axiomatic that the number of species 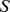 grows approximately with the sampled area 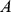 as 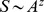, where 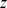 denotes the species–area exponent. If the relative species abundances follow a lognormal distribution, and each area is a random sample from the larger population, then according to Preston 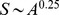, i.e., 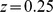 [27]. While the canonical relationship of Preston has not been evidenced by empirical measurements, the values of 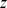 are rather conservative, and the great majority of the reported values are within the range 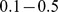 [26]. From a different perspective, MacArthur and Wilson have proposed that the number of species in a given region arises from a balance between immigration and local extinctions [28]. These ideas have been further developed: dispersal and speciation have been incorporated into a spatially explicit model aiming at explaining the observed patterns of species–area relationships [18], [19]. This model is a version of the voter model, primarily conceived for studying the spreading of opinions in a social system [17].

The voter model with mutation as defined by Durrett and Levin [18], and used here, is represented on the space by a two-dimensional integer lattice of size 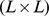, which is then divided into square cells (sites), each one being occupied by at most a single individual. The model assumes reproductive equivalence of species, regardless of a species’ identity, a premise of Hubbell’s unified neutral theory of biodiversity and biogeography [19]. At each time step an individual chosen at random dies. This empty location is then, with probability 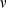, filled by a new species (speciation event), or else it is filled by a species chosen from its *neighbourhood*, which comprises the four cells that are orthogonally adjacent to the site of interest (the *von Neumann neighbourhood*). In the latter event, the four neighbouring cells are equally likely to contribute offspring to occupy the empty location. Note that as soon as an individual is eliminated, its location is immediately filled up. Therefore, each time step corresponds to a death event, and the parameter 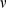 is the speciation rate in units of the death rate (for a detailed description of the model, see [18]). In fragmented landscapes, as studied here, only the suitable habitats in the neighbourhood contribute to the recolonization of an empty location.

The species are indexed by a real number in the interval 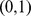, and each new species is labelled by a randomly chosen number from this interval not duplicating an existing index. A cell’s being in state 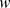 indicates that it is occupied by one individual of species 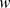, whereas the state 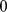 denotes an unsuitable habitat. The population evolves according to the above dynamics until the system reaches an equilibrium regime. Although the model provides some dispersal among next-nearest cells, it is feasible to allow long-range dispersal [29]–[32], as we will briefly discuss in subsection 3.4. For the sake of completeness it is important to mention that our model assumes a finite landscape with closed boundary conditions.

#### Long-range dispersal

The aforementioned model can be generalized with a dispersal rule enables individuals to disperse over larger distances than the next-nearest cells. In Results section we briefly discuss the results for this generalized model, in order to address the role of immigration in the patterns of the species–area relationships.

Once again, at each time step, a randomly chosen individual in the grid dies and gives rise with probability 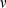 to an individual of a new species, and otherwise is replaced by one of the species which are located inside a square dispersal kernel of linear size 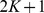 centered on the empty location. All individuals in the dispersal kernel have the same probability of sending offspring to the empty site, i.e., 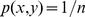, where 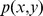 is the probability an offspring is sent from 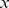 to 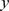 and 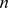 denotes the number of suitable habitats, excluding the empty location, in the area of size 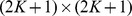. This is the simplest form of dispersal kernel, though Rosindell and Cornell have shown that the different forms of dispersal kernels are equivalent provided that the averaged square dispersal distances are the same [29].

### The Coalescence Method

Instead of performing extensive forward simulations in time, a powerful approach known as the coalescence method can be used to efficiently produce equilibrium configurations in the neutral model [21]. According to this method, an infinite-time equilibrium configuration can be reached by tracing backwards in time the evolutionary trajectory of the population. The key idea of the method is to construct the genealogy of the entire population until the most recent common ancestor is found. The problem is mapped onto a system of coalescing random walks whereby each individual performs a random walk over a finite lattice. The time at which the walkers coalesce into a single walker is related to the consensus time of the voter model. In addition to coalescence events, the approach also considers the random extinction of walkers at rate 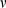, equivalent to speciation events in the forward approach. For more details about method, see [21], [30].

When nearest neighbour dispersal is assumed, as in the original voter model, unconnected clusters of suitable habitats have independent genealogies. Under this circumstance, one way to minimize the computational cost is to apply the Hoshen–Kopelman algorithm [33] so that all clusters are properly recognized, and we then perform independent runs of the coalescence approach to each cluster independently. Here, *cluster* refers to a contiguous structure of occupied sites which is surrounded inside and outside by vacant regions of irregular shapes and areas, and so each cluster is disconnected from every other cluster in the lattice. The Hoshen–Kopelman algorithm [33] was an important breakthrough for cluster analysis in percolation theory. It owes its success to its linear time and superior space computational complexities in terms of lattice size [34], [35]. The Hoshen–Kopelman algorithm probes the grid, looking for occupied cells. The purpose of the algorithm is to assign a cluster label to each occupied cell, so that cells belonging to the same cluster get the same label. The easiest situations occur either when a cell is isolated and hence receives a new label, corresponding to a new cluster, or when there is a single occupied cell in its immediate neighbourhood, and in this case it receives the same label as its neighbour. In the case the focal cell has more than one occupied neighbouring cell, one ascribes the lowest-numbered cluster label. Occupied neighbouring cells that are identified by different cluster labels indeed belong to the same cluster, and therefore as a final stage, the algorithm merges these clusters into one cluster.

### Measurement Procedures

This study mainly concentrates on measurements of biodiversity, within a sampled area, that correspond to the number of species in the area regardless of their commonness or rarity. In the simulations, the length of the side of the system is kept fixed, 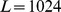. We perform measurements of the 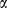-diversity, which means that to assess the spatial pattern of the species distribution, one increases the coverage of the system (which has a constant size) by using larger and larger sampled areas. The species–area relationships are thus obtained through dividing the grid into several sublattices, or regions, and then taking averages over all the distinct regions. Regions entirely composed of unsuitable sites are not taken into account. Ensemble averages are performed over 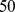 independent runs.

## Results and Discussion

**Figure 1:**
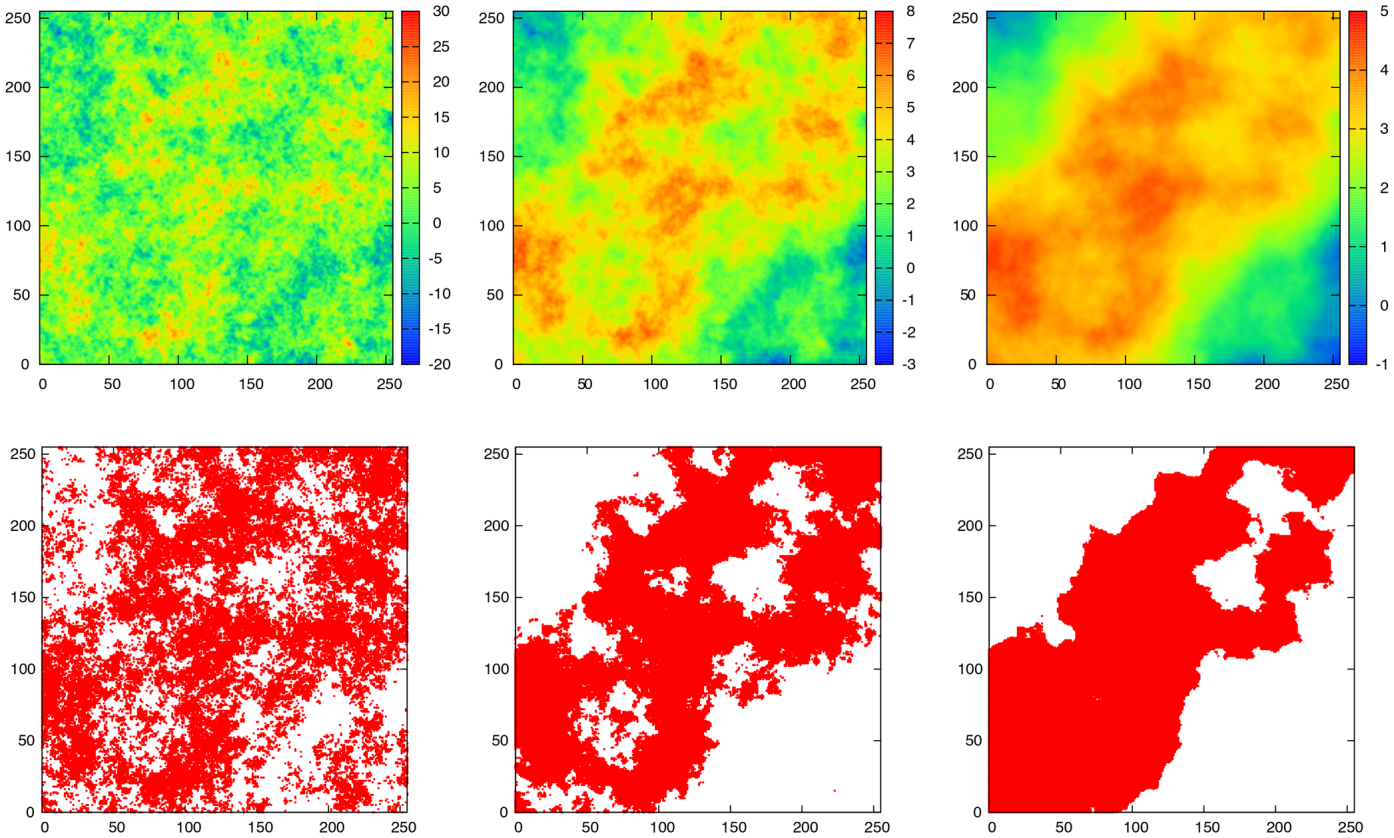
Instances of fragmented landscapes with varying degree of spatial autocorrelation. In the left panels the Hurst exponent is set at 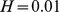, while for the middle and right panels its value is set at 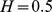 and 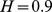 respectively. In the upper panels the sites in a 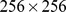 grid are are color-coded according to an elevation scale 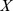, with scale identifier shown along the right side bar. To each site in the grid corresponds a height, as provided by the fractional Brownian motion (fBm) algorithm. The lower panels are obtained from their corresponding configurations in the upper panels by fixing the fraction of suitable habitats at 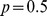.

The lower panels of Figure 1 depict three typical configurations of fractal landscapes generated in these simulations, which differ in the amount of spatial autocorrelation. The suitable habitats are represented by black sites. From left to right, 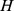 increases from 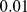, a situation which characterizes a highly fragmented landscape, to 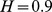, where large connected domains of populated sites are obtained. These three configurations of black and white sites derive directly from the upper panels, where the points in the grid are represented in greyscale by an elevation scale 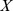 with scale identifier shown along the right side. The fraction of suitable habitats 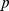 then settles a critical height value 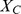, and thereby assigns the value one to every point with elevation 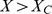, denoting a suitable habitat, whereas points with elevation values 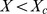 are set to zero, this denoting an unsuitable habitat.

### Biodiversity Measurements

**Figure 2:**
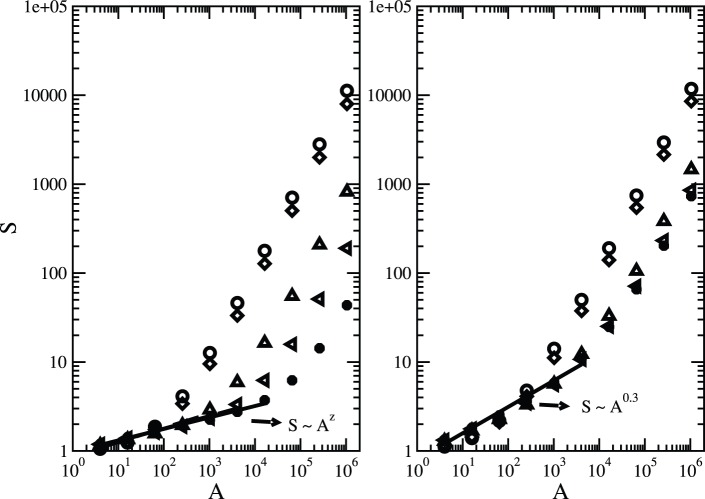
Effect of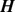 on biodiversity levels. Species-area relationships for different values of the Hurst exponent 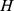 and fixed fraction of suitable habitats 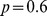. The parameter values are 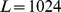; Hurst exponent: 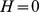 (circles), 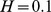 (diamonds), 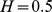 (triangles up), 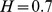 (triangles left) and 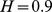 (stars); and speciation rate: 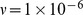 (left panel) and 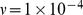 (right panel). The data points represent an average over 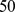 independent runs. The error bars are smaller than the symbols.

Figure 2 shows the average diversity of species as a function of the area, 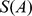, for lattices with a side of length 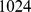, and five distinct values of the Hurst exponent. In the figure, the same value of occupation probability for the habitat sites is used 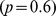, and fixed values of the speciation rates are considered: 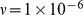 (left panel) and 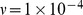 (right panel). Except in the range of very small areas, the observed biodiversity 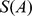 increases when 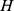 decreases, for fixed values of area: this is due to the fact that if 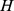 is low, the population is dispersed over a large number of unconnected domains, which also means that they are geographically isolated from each other. Another important feature is that as 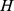 decreases, it enhances the chance that different parts of a given cluster become linked through narrow corridors. In this way, because one has nearest neighbour dispersal, it is very likely that one species blocks the corridor, making it impassable to any other species [30]. On the other hand, for larger 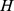, closer to unity, corresponding to smoother landscapes, we get a greater effective connectivity of the habitat, with a small number of fragments, and thereby the number of species shrinks for a fixed area. This effect occurs mainly because the onset of the linear regime, which takes place in broad scales, is shifted to larger sampled areas as the landscape becomes more clumped.

The same plot demonstrates a nontrivial dependence, 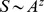, in the range of small and intermediate sampled areas, which is achieved for intermediate and large values of the clumping parameter 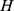. The validity of the scaling 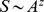 then extends over a broader interval of areas as 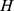 approaches unity. The estimated 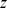-values are 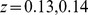 and 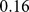, when 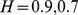 and 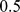, respectively. Larger values of 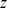 are estimated for an upper speciation rate (right panel), 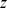 being in the range 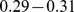.

**Figure 3:**
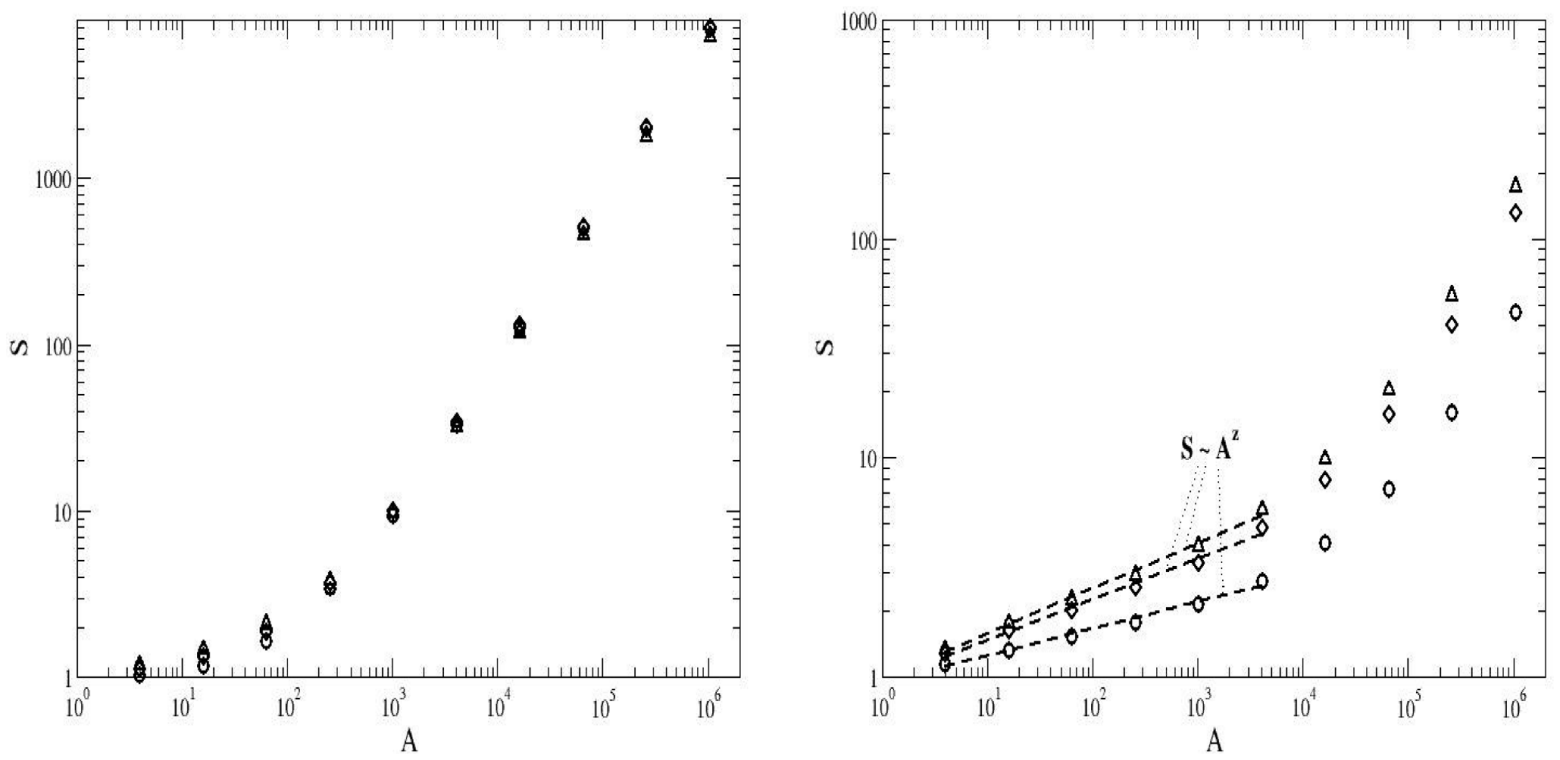
Change of the biodiversity levels with the occupation probability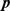. The parameter values are lattice size 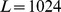, speciation rate 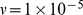, and Hurst exponents 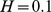 (left panel) and 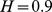 (right panel). The occupation probabilities are 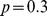 (circles), 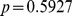 (triangles) and 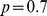 (diamonds). The data points represent an average over 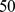 independent runs. The error bars are smaller than the symbols.

The non-trivial relationship 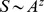 is especially hard to observe in highly fragmented landscapes since as the fraction of suitable habitats approaches the critical threshold 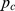, the power-law regime becomes constrained to a very narrow range of sampled areas [30]. When the fraction 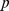 of suitable habitats is below the critical threshold, the landscape becomes fragmented into a large number of domains. It is important to point out that the critical threshold depends on 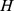, shifting to lower 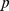 as 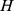 grows. As such, it is expected that the power-law regime will persist over a broader range of 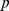 for more clumped landscapes [24]. Furthermore, Figure 2 evinces a dispersion of the biodiversity values with 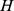 as the speciation rate increases. That is, the 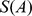 curves tend to collapse, irrespectively of the value of 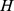 for sufficiently high speciation rates, reflecting the irrelevance of the landscape details in this particular domain of the speciation rate. A collapse of the species–area curves can also be attained in the state of high fragmentation, as indicated in Figure 3. We notice that the onset of the linear regime occurs at the beginning of the scale. In this case, it is not possible to describe the dependence of 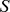 on 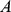 by a non-trivial scaling, 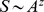. In fact, a non-trivial scaling, 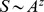, is achieved when a higher degree of clumping is considered, as shown in Panel (b) of Figure 3. In these instances, the best fittings provide 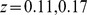 and 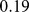, when 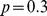, 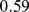 and 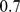, respectively. We thus see a slight increase of 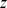 with 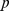, which is not due to any substantial change of the spatial pattern of the landscape, but instead is due to a change in the number of habitats. We also note that higher levels of biodiversity (larger 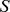) are sustained as the amount of habitats raises in highly clumped landscapes. A similar outcome is seen in a multispecies contact process (MCP) [36], whereby fragmentation stems from the internal dynamics and not from the structure of the landscape, being the state of the sites (vacant or occupied) a dynamical feature. In that model, individuals also tend to be clumped since the colonization of vacant sites depend on the occupation of their neighborhood. In their study, Cencini et al. [36] show that as the expected occupation level increases the biodiversity levels approach the outcome of non-fragmented habitats.

### The Distributions of Species Abundance

**Figure 4:**
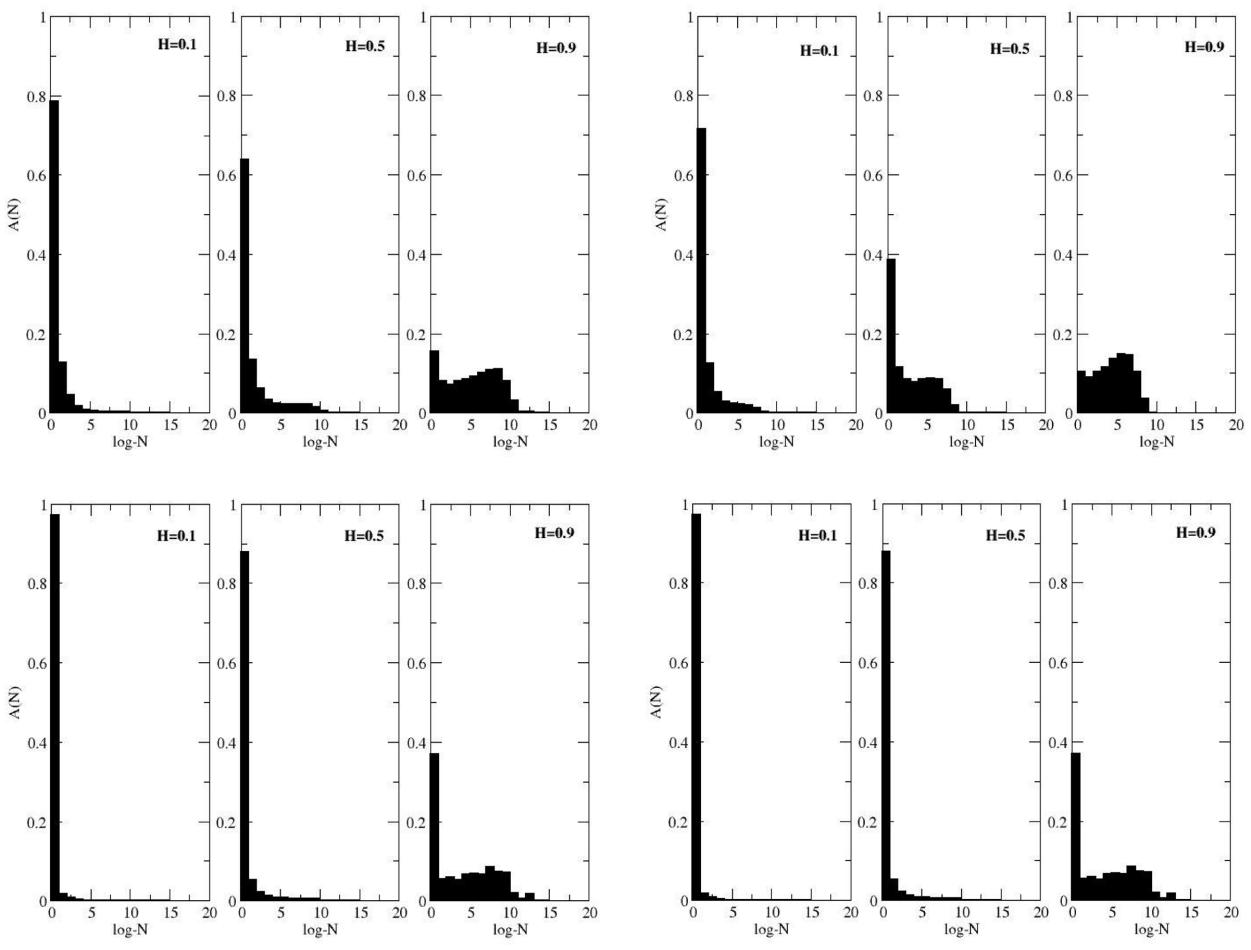
Species abundance distributions. Upper panels: the parameter values are 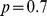, 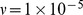 (left set of panels) and 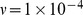 (right set of panels). Lower panels: the parameter values are 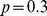, 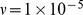 (left set of panels) and 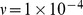 (right set of panels). The Hurst exponents are indicated in the figures.

The coalescence method affords us not only the possibility to obtain the number of species within a sampled area, but also provides information about their distribution across the community. Making use of this information, complementary achievements about the behaviour of the neutral community with the features of the landscape can be obtained, such as the distribution of species abundance, which tells us how common or rare a species is relative to other species. In Figure 4, we have the number of species, 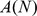, plotted for different abundance intervals, i.e., the number of individuals 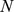 (log-bins, logarithms are to base 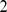). The results are shown for different values of the Hurst exponent. The division of bins follows the procedure used by Chave [37], [38], where the bins encompass the abundances 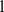, 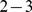, 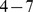, 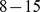, and so on. The qualitative pattern is nearly the same when comparing the panels, despite the change in the occupation number 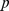 and speciation rate 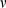. This figure suggests that the special case 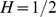 reflects a separation between two different regimes of 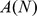. For 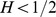, the distribution 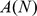 is unimodal and decays hyperbolically with 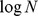, while for 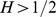, the abundance presents a bimodal aspect, with two maxima for populations of different sizes. In addition to the dependence on 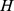, it is worth mentioning that the small population peak clearly shortens with 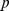 and also, to a minor extent, with 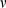. In all these plots, the sample size is equal to the total community size so that the statistics takes into account all the existing species.

### Fractal Dimension of Species Distribution

A question of general interest which has not yet been examined is how each species fills up the suitable habitats from the point of view of fractal dimension. Although this is a complex undertaking for the entire community of species, at least we can ask how it is distributed in our ecosystem model for the principal dominant species in terms of the basic parameters of interest. In particular, it is opportune to know about the fractal dimensions 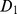 and 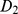 of the sets where the first and the second dominant species are concentrated. In order to answer this question, we performed an extensive statistical analysis of these dimensions for different values of the parameters 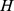, 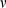, and 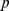. To accomplish this task, we used the box-counting method [39]. In this method, we count the number 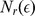 of square cells of size 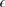 needed to cover all the sites where the dominant species of a particular rank 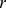 is distributed. In the scaling region the fractal dimension 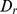 and 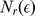 are simply related by 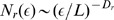, where 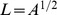.

Whereas the SAR relation involves two macroscopic quantities, namely the number of species 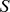 and the area 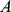 where such species are distributed, the box-counting relationship 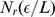 introduces a microscopic analysis which complements that macroscopic description. Thus if we know the set 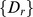 of fractal dimensions associated with the supports of the distribution of each species, we have in the scaling region the sum rule



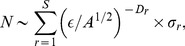
(3)for fixed length scales 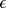 and 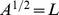, with 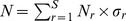 being the total number of individuals irrespective of species within the area 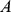, and 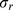 the density of species 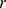 at the same resolution (i.e., the average number of individuals of species 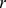 within boxes of area 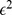).

If each species is distributed with a two-dimensional support, i.e., if for each species 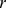, 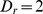 is a constant, the last result must reduce trivially to
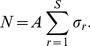
(4)


On the other hand, if different species are not associated to the same fixed dimension 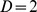 of their support, equation (4) does not follow and we need to consider the more general equation (3). In this way, we believe that such types of measurements of 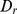 are important. They could be implemented in field research or in controlled lab experiments with a small number of species of microorganisms competing for resources in a culture medium of area 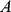.

**Figure 5:**
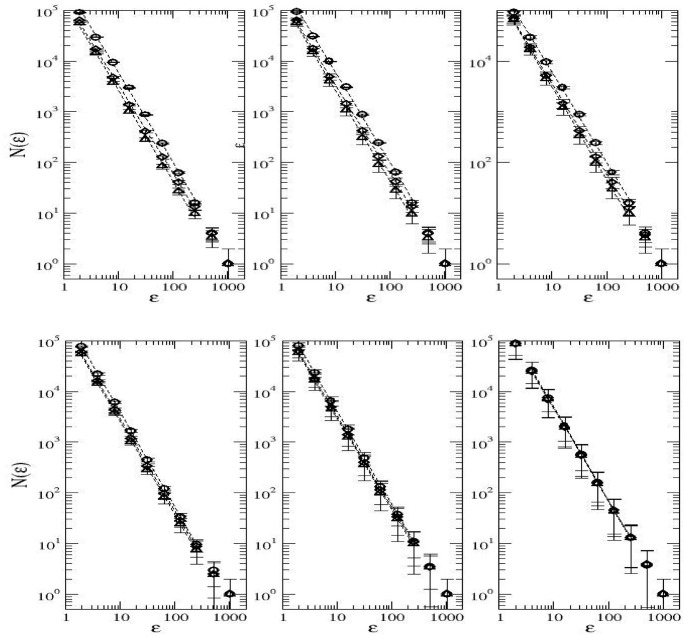
Measurement of the fractal dimension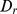 of the spatial distribution of the commonest species. 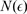
 versus 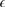. The parameter values are 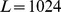, speciation rates 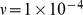 (left panels), 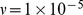 (middle panels), 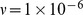 (right panels). In the upper panels the fraction of suitable habitats is set at 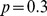, whereas in the lower panels 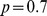. The symbols denote different values of 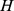: 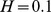 (circles), 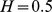 (diamonds) and 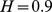 (triangles). The data points represent an average over 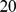 independent runs. The error bars correspond to one standard error.

Typical plots of 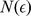, which represent averages over 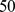 independent runs, for different sets of parameter values are shown in Figure 5. It can be observed that all these plots exhibit a clear scaling behaviour over three orders of magnitude of the side length. Table 1 shows the variation of the fractal dimensions 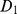 and 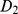 with 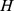, 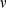, and 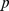. It indicates that all the values of 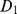 are confined within the interval 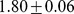, i.e., the relative dispersion of 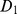-values is inferior to 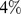. The values of 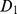 are essentially independent of 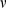 and 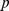 provided that 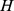 is constant. The dimension 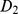 found in our simulations is significantly smaller than 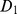. Unlike 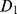, 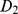 clearly decays as the speciation rate grows. That is, unlike the behaviour exhibited by 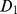, the values of 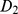 are dependent on 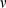, although not very dependent on 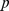, for 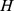 constant. Actually, all these measurements lack empirical evidence, mainly due to the difficulties involved in obtaining field data in ecosystems for the abundance versus the fractal dimensions. There are only a few empirical measurements that try to quantify the correlation between the degree of aggregation (which is not the same as the fractal dimension) and the species abundance in tree species [40], [41]. Though, even if these relations between the abundance of the species and their fractal dimensions are only be warranted within the framework of the neutral dynamics model, which serves the purpose of a null model, it could shed new light on basic aspects connected with the practical usefulness of the neutral theory.

**Table 1:** Fractal species distribution.

	*H* = 0.1	*H* = 0.5	*H* = 0.9
*v* = 1×10^−4^ ; *p* = 0.3	1.78 (1.49)	1.74 (1.50)	1.80 (1.51)
*v* = 1×10^−5^ ; *p* = 0.3	1.79 (1.55)	1.74 (1.62)	1.80 (1.62)
*v* = 1×10^−6^ ; *p* = 0.3	1.78 (1.69)	1.77 (1.69)	1.82 (1.73)
*v* = 1×10^−4^ ; *p* = 0.7	1.86 (1.48)	1.82 (1.48)	1.84 (1.47)
*v* = 1×10^−5^ ; *p* = 0.7	1.85 (1.58)	1.80 (1.59)	1.81 (1.60)
*v* = 1×10^−6^ ; *p* = 0.7	1.84 (1.72)	1.81 (1.73)	1.82 (1.73)

The fractal dimension of the distribution of the first (second) commonest species.

### Long-range Dispersal

**Figure 6:**
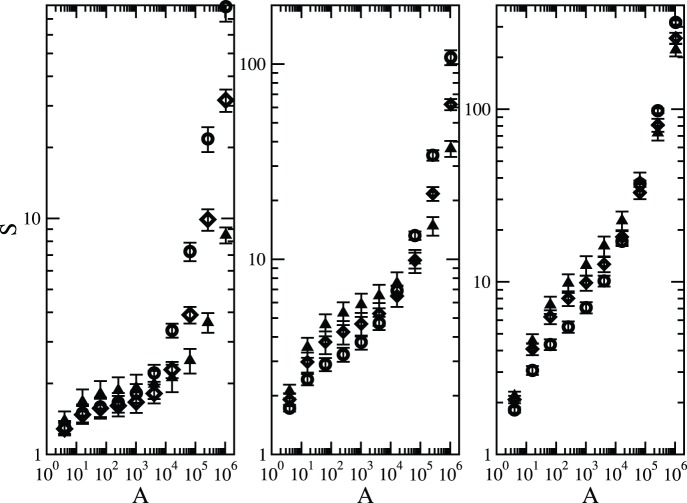
Effect of dispersal on the species-area relationship. Number of species 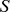 is plotted against area for 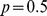 and dispersal parameter 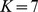 and distinct values of the clumping parameter 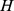: 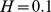 (open circles), 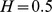 (open diamonds) and 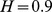 (filled triangles). Left panel: 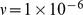, middle panel: 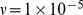 and left panel: 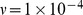. The data points represent an average over 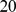 independent runs. The error bars correspond to one standard error.

Figure 6 compares the species–area relationships for a fixed speciation rate and varying values of the clumping parameter 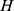. As expected, when further neighbour dispersal is allowed, there is a departure from a biphasic to a triphasic shape. This is owing to the appearance of a second crossover point, which delimits the first phase for small areas from the intermediate phase where the power-law regime 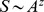 holds. This crossover point occurs around areas of size 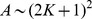 where dispersal is the dominant mechanism [31]. The triphasic scenario becomes less prominent as the speciation rate increases. These curves are steep at fine and broad scales and shallow at intermediate scales [19], [26], [42]. This triphasic pattern is a quite universal aspect of species–area curves when viewed over many orders of magnitude of area [19], [26]. The theoretical explanations invoke random sampling and broad-scale dispersal limitation as the main determinants of this relationship [19], [26].

One important feature of the plots in Figure 6 is that for low and intermediate areas, smoother structures (high 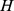) can harbour a higher number of species, whereas very fragmented landscapes sustain a greater number of species in broad scale. This is somewhat expected since the ecological processes are uncorrelated in the broad scale because of limited dispersal, thus the enlargement of the coverage area implies the merging of distant locations with very distinct species composition. Therefore, in this continental regime, as it is called by Hubbell (2001), the number of species grows approximately linearly with area. This aspect is even stronger in extremely rough landscapes. On the other hand, in fine and intermediate scales, a more compact region is less susceptible to local extinctions due to ecological drift since these structures exhibit a greater effective connectivity.

**Figure 7:**
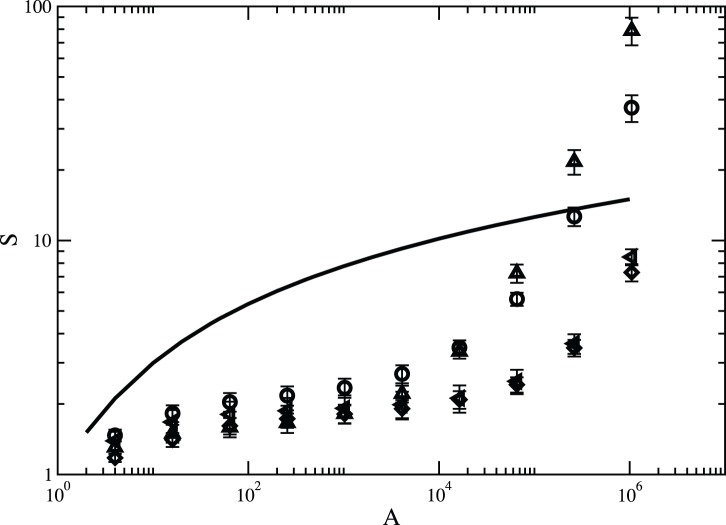
Species-area curves for different values of dispersal parameter. Number of species 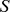 is plotted against area for 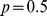 and speciation rate 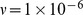. The symbols denote diferrent set of parameter values : 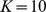 and 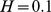 (circles), 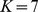 and 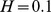 (triangles up), 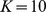 and 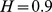 (diamonds), 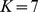 and 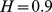 (left triangles). The line is the theoretical prediction for the species-area curve if dispersal is not limited as given by Eq. (5). The data points represent an average over 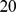 independent runs. The error bars correspond to one standard error.

Figure 7 shows the species–area curves for different values of the dispersal parameter 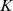. For very clumped landscapes 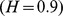, one does not observe any strong influence of the area of the dispersal kernel on the observed values of species number 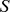. On the other hand, in rough landscapes 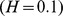, one observes that dispersal limitation reduces the biodiversity in the fine and intermediate scales, mainly because increasing values of 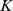 permit connecting regions supposedly unconnected, a fact that weakens the role of ecological drift and so reduces local extinctions. As aforesaid, in a broad scale, the ecological processes are uncorrelated and there is a merging of regions with dissimilar species composition. The dissimilarity is certainly enhanced in rough structures. In the same plot, the comparison with the theoretical prediction for the species–area curve under the assumption of no dispersal limitation, as demonstrated by Hubbell [19], is made. According to Hubbell, the expected number of species 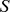 is given by
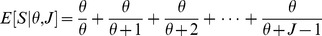
(5)where 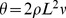 is the fundamental biodiversity number, and 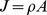. The quantity 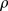 corresponds to the density of individuals, which here is the same as the frequency of suitable habitats 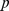, 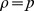. It is clearly seen that the assumption of no dispersal limitation greatly enhances the number of species, especially for small and intermediate areas, and then its rate of growth slows down in the broad scale. For large 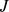, Eq. (5) provides that the number of species depends logarithmically on the sample size [19], [43].

### Conclusions

There has been a long-standing debate in conservation biology about the effects of habitat fragmentation and loss on biodiversity [8], [44], [45]. This issue has been addressed in the present paper within a steady state perspective. The very diverse conceptualizations and measurements of habitat fragmentation in the landscape ecology literature has been a cardinal difficulty for unifying and producing concise conclusions about the role of habitat fragmentation in changes in the composition of ecosystems.

For a deeper understanding of the role played in biodiversity by interplay between landscape structure and its ecological response, the present paper has employed a more realistic spatial pattern for habitat distribution through the use of a different landscape model, whereby the fragmentation of the landscape can be controlled by the parameter 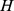, which tunes the degree of spatial autocorrelation among adjacent cells. Our choice relies on the class of fractal landscapes, which have long been recognized as a very useful tool in the realistic rendering and modeling of natural phenomena [15]. This design allows us to address the relationship between the degree of habitat fragmentation and the strength of the biodiversity response. Naturally, these neutral models have limitations and are not able to portray all the complex spatial patterns present in real landscapes.

In order to simulate fractal landscapes with tunable properties, we employed the fractional Brownian motion model of Mandelbrot and Van Ness [25]. The nontrivial biodiversity–area regime 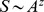 was identified and the dependence of the exponent 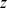 on 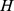, the speciation rate 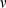, and the occupation probability 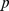 was obtained. There is a slight propensity for increased values of the species–area exponent 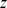 as the landscape becomes more fragmented. When dispersal is constrained to the next-nearest neighbours, as originally assumed by Durrett and Levin [18], a biphasic scenario emerges. The crossover point which delimits the fine and intermediate scales from the broad scales is very sensitive to the speciation rate 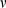, the fraction of suitable habitats 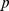, and the aggregation parameter 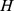. Long-range dispersal changes considerably the patterns of the species–area curves, which now exhibit a triphasic behaviour. The additional crossover point now delimits the fine and intermediate scales and occurs around areas of size 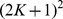, where dispersal is the prevailing ecological mechanism [30]–[32]. Thus, the shape of the species–area relationship is also clearly affected by species characteristics, more especially their gap-crossing abilities which can vary by orders of magnitude. For example, species-area relationships for birds clearly display a triphasic pattern [26]), whereas flowering plants conform more to a biphasic pattern, very similar to those reported in our simulations [26].

Furthermore, the dependence of the abundance of species as a function of the population as well as the dependence of the fractal dimensions 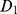 and 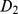 of the support where the first and second dominant species are distributed were examined for different values of 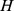, 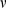 and 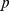. The extension of this last type of analysis within our model for the third, fourth, 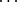, rth dominant species is of great interest because we know that different species use different kinds of habitat, and different species require different amounts of habitat for their persistence [8]. A complete analysis of the model studied here in the space of parameters (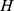, 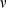, 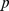) is far beyond the scope of the present paper and in this sense we urge that a more systematic investigation be made to evaluate all the potential uses of this model for taking into account all the effects of habitat fragmentation on the biodiversity of real systems.
